# UNMET SERVICE NEEDS AND HOME AND COMMUNITY-BASED SERVICE USE IN RURAL AND URBAN DEMENTIA CAREGIVERS

**DOI:** 10.1093/geroni/igae098.3535

**Published:** 2024-12-31

**Authors:** Christina Miyawaki, Angela McClellan, David Russell, Erin Bouldin

**Affiliations:** University of Houston, Houston, Texas, United States; University of Houston, Houston, Texas, United States; Appalachian State University, Boone, North Carolina, United States; University of Utah, Salt Lake City, Utah, United States

## Abstract

The lack of resources and services for caregivers of people with dementia (PWD) in rural areas in the U.S. has been well-documented. However, less research has explored the conditions of caregivers in urban areas. The purpose of this study was to identify the unmet caregiver service needs of urban caregivers of PWD, explore factors associated with their service use, and compare the outcomes with those of rural counterparts. Guided by Andersen’s theory of health service use, we interviewed 20 caregivers in rural Western North Carolina and 18 caregivers in urban Houston, Texas. Using the same interview guide, we inquired about their service needs and use, and contextual factors that facilitate their service use, and conducted a thematic analysis of the interviews. Interviews revealed that rural caregivers wished for more information and caregiver support whereas urban caregivers searched for information until they found satisfactory solutions. Rural caregivers expressed guilt about using services because they were limited and zero-sum while urban caregivers sought to assist their fellow caregivers through sharing available resources. Urban caregivers needed more culturally tailored services while rural caregivers’ cultural needs were more place-specific. Increasing options for online and virtual support groups and expanding the reach across the U.S. might address the unique concerns of rural caregivers. Developing ethnic-specific resources for their diverse population and PWD represents a priority among urban caregivers. Racially/ethnically diverse health professionals, including nurses and social workers with multi-linguistic skills, are needed to meet the needs of racially/ethnically diverse patients and caregivers in urban areas.

